# Effects of Biochar and Sepiolite on Pb and Cd Dynamics in Contaminated Soil with Different Corn Varieties

**DOI:** 10.3390/toxics13020127

**Published:** 2025-02-09

**Authors:** Peiyi Zeng, Muqing Yang, Shujuan He, Ying Kong, Xian Zhu, Zitao Ma, Min Wu

**Affiliations:** 1 Faculty of Environmental Science and Engineering, Kunming University of Science and Technology, Kunming 650500, China; peiyizeng529@163.com; 2 Yunan Research Academy of Eco-Environmental Sciences, Kunming 650034, China; yangmuqing0414@163.com (M.Y.); ynhsj1989@126.com (S.H.); zjg29934826@163.com (Y.K.); zhuxianyunduo@163.com (X.Z.); 13908777720@163.com (Z.M.); 3 Yunnan National Engineering Research Center for Control &Treatment of Heavy Metal Pollution, Kunming 650034, China

**Keywords:** heavy metals, biochar, sepiolite, biological accumulation coefficient, transfer coefficient

## Abstract

Biochar can stabilize heavy metals in soil and inhibit their accumulation in plants as a soil amendment. Sepiolite has also shown good effects in the remediation of soil heavy metal pollution. In this study, biochar, sepiolite, and biochar–sepiolite combined amendments were used to evaluate the accumulation of cadmium (Cd) and lead (Pb) in soil by 29 corn varieties. The concentrations of Cd and Pb in corn fruits were the lowest (Pb: 0.11 mg/kg, Cd: 0.06 mg/kg). There was a significant difference (*p* < 0.05) in Pb and Cd accumulation in the roots, stems, leaves, and fruits in the 29 corn varieties. The BCF and TF values of Pb and Cd in the 29 corn varieties were different, and Pb is more likely to accumulate in the roots, Cd is more likely to accumulate in the leaves, and neither heavy metal is easily translocated to the corn fruits. The combination of biochar and sepiolite creates an environment conducive to the retention of heavy metals in the root zone, effectively reducing the risk of heavy metal contamination in the edible parts of the plants. After considering various factors, such as environmental adaptability, we recommend using sepiolite and biochar combined as a soil amendment material and planting the WG1790 variety. Field experiments are needed to verify the effects. These results provide scientific evidence and new strategies for the selection of corn varieties and soil amendments.

## 1. Introduction

Since the Industrial Revolution, increases in industrial scale have led to rising levels of heavy metals in the environment, and heavy metal pollution has received widespread attention [1,2]. Lead–zinc smelting activities release a large amount of heavy metal particles into the air during the production process [3]. These particles combine airborne particulate matter and settle on the ground, resulting in severe soil pollution. And heavy metals that enter the soil not only affect the yield and quality of crops, but can also transfer into the food chain through plant tissues, exposing humans to contaminated food [4,5,6]. According to previous studies, prolonged exposure to food contaminated with heavy metals can damage the brain, liver, kidneys, and nervous system, leading to oxidative stress, cancer, cardiovascular diseases, and other health issues, posing a serious threat to human health [7,8]. Therefore, removing heavy metals from the soil is of great significance for ensuring food safety and protecting human health.

Adding amendments to the soil can effectively reduce the likelihood of heavy metals entering the food chain [9]. Park et al. [10] found that the addition of soil amendments to contaminated soils can effectively stabilize heavy metals. Amendments reduce the activity of heavy metals and limit their accumulation in plant tissues to some extent by physical entrapment, precipitation with minerals, exchange with cations, complexation with oxygen-containing function groups, and coordination with π electrons [11,12,13,14,15,16,17]. Various amendments have been developed and used for the remediation of heavy metals in soil, including zeolite, lime, phosphate fertilizers, and red mud [18,19]. However, these amendments lack cost-effectiveness and efficiency, which limits the process of soil remediation [20]. Therefore, exploring an economical and efficient soil remediation method holds significant practical importance.

Biochar, as an economical and environmentally friendly solid carbon material, has gained widespread attention [21]. It can stabilize heavy metals in the soil and, as an amendment, inhibit their accumulation in plants [22,23,24,25]. Li et al. [26] found that the addition of biochar to the soil reduced the mobility of heavy metals, resulting in a 45–49% reduction in heavy metal accumulation in plants. Sepiolite is also an excellent soil conditioner with strong surface adsorption and ion exchange capabilities. China is one of the countries rich in sepiolite [27], and studies have applied sepiolite in the remediation of heavy-metal-contaminated soils, achieving good results in recent years [28,29]. There are few studies on the remediation effect of these two composite materials on metal-polluted land, and more studies were needed [30,31].

Corn exhibits a low heavy metal uptake ability with minimal accumulation of heavy metals in the edible parts, meaning that it is considered a low-accumulation crop (the concentration of heavy metals remained below the permissible limit (Pb:0.2 mg/kg; Cd:0.1 mg/kg) and had low BCF and TF values (<1)) [32,33]. In total, 29 corn varieties from Yunnan Province, China, were selected in this study. The accumulation status of Cd and Pb in corn varieties was evaluated under four conditions: biochar amendment, sepiolite amendment, biochar–sepiolite combined amendment, and no amendment. This study calculates and analyzed the bioconcentration factor, transfer factor, heavy metal extraction efficiency, and accumulation of Pb and Cd under different conditions. This research verifies the improvement effect of biochar and sepiolite amendments on Pb-Cd-contaminated soils and identifies corn varieties with stronger heavy metal absorption capabilities in different conditions. These results are expected to provide new insights for the remediation of Pb-Cd-polluted soils and offer data support for relevant policy formulation.

## 2. Materials and Methods

### 2.1. Study Areas and Regional Characteristics

**Figure 1:**
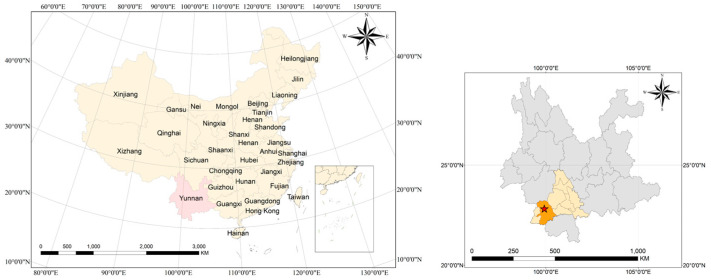
The location of Lancang Country and field trial.

This study area was in Lancang Country, southwestern Yunnan Province, with geographic coordinates from 22°01′ N to 23°16′ N and from 99°29′ E to 100°35′ E. Lancang County covers an area of 8733 km^2^, making it the second-largest county in Yunnan in terms of area, and it is rich in mineral resources (Figure 1). The Lancang Pb-Zn mining area was once one of six key heavy metal prevention and control areas in Yunnan province, highlighting the importance of addressing soil pollution issues. The Lancang Pb mine area is a typical heavy-metal-polluted land.

### 2.2. Cron Variety Selection

Based on the *Lancang County Economic and Social Statistical Yearbook*, the 2020 Rural Major Products Production Table released by the Lancang County Statistics Bureau, on-site investigations, and interviews, the main crops cultivated are corn, sugarcane, and rice in Menglang Town, Lancang County. Considering the limited area for tea, coffee beans, vegetable crops in the project area, local planting habits, and farmers’ willingness, corn was selected as the primary crop for screening low-accumulation high-biomass varieties. Considering the region’s environmental and pollution characteristics, the corn variety screening was conducted in accordance with the primary varieties promoted within the project area, along with the low-accumulation corn varieties identified in previous studies. The selected varieties and details are shown in Table S1.

### 2.3. Experimental Design

#### 2.3.1. Corn Cultivation

On 6 June 2022, a field trial was conducted within 5 kilometers northeast of Yunnan Lancang Pb Mining Co., Ltd., in Lancang County, Pu’er City. The field trial included 179 corn variety screening plots, with a planting density of 35 cm × 35 cm; the soil texture used in the experiment was red clay. We randomly planted 29 corn varieties in the plots, with 3 seeds sown in each planting hole. For base fertilizer application, 500 kg of well-decomposed organic fertilizer or 100 kg of compound fertilizer was used per acre. The characteristics of the fertilizer are shown in Table S2. For the top dressing, 30 kg of urea and 20 kg of compound fertilizer were applied. It was found that applying the top dressing twice yielded the best results: the first application during the jointing and booting stage (accounting for two-thirds of the total top dressing), and the second application during the grain filling stage (accounting for one-third of the total). Most of the relevant studies in China focused on the growth of cultivated crops and food security issues. In addition, during the field experiment, tillage and other agricultural operations were consistent for all samples, the tillage layer soil was evenly mixed by applying conditioners, and the soil particle size state at each point of the experimental sample was consistent. Therefore, in this study, it was considered that soil particle size characteristics did not have a major influence on the experimental results, and they were not included in the consideration of the soil characteristics tested.

#### 2.3.2. Sample Collection and Detection Analysis

The corn matured in mid-September 2022. After being naturally dried in the field, mature corn and soil samples (0–20 cm) were collected on 30 September 2022, using a five-point sampling method for the sample of corn and soil. The corn plant samples were divided into four parts, roots, stems, leaves, and grains, and were sequentially washed with tap water, Na_2_-EDTA, and deionized water. The cleaned plant materials were placed in an oven at 105 °C for 30 min, followed by drying at 75 °C for 48 h. After drying, the dry weights of each part were recorded and the samples were ground using a stainless-steel grinder and sieved through a 100-mesh sieve. Soil samples were air-dried in a greenhouse, and plant roots and stones were removed before passing through a 100-mesh sieve. The sieved soil and corn samples were stored in plastic sealed bags for heavy metal content analysis (dry weight).

#### 2.3.3. Analysis Methods for Corn and Soil Samples

Soil was digested using aqua regia and HClO_4_, while plant samples were digested with HNO_3_-H_2_O_2_. The contents of Cd and Pb in the digested solutions were determined using flame atomic absorption spectrometry (AA240FS, Varian, Las Vegas, NV, USA). As content in the digested solutions was determined using atomic fluorescence spectrometry (AFS, AF-610D) after reduction with thiourea and ascorbic acid at a certain dilution ratio, the same method was used to determine the standard sample, and the recovery rates for Cd, Pb, and As were between 93% and 98%, meeting the quality control requirements for heavy metal detection. Weigh 10.00 g of soil sample into a 50 mL beaker and add 25 mL of water. Seal the container with a sealing film and stir with a magnetic stirrer for 2 min, then let it stand for 30 min. After that, gently shake the container to form a suspension, and measure the pH using a pH meter (FE28). More details are shown in Table S3 [34,35,36,37,38,39,40,41,42].

### 2.4. Data Analysis

The experimental data were processed using WPS Excel, and variance analysis (ANOVA) was conducted using SPSS 19.0. Multiple comparisons of means were performed using Tukey’s HSD (Honestly Significant Difference) method. Cluster analysis was conducted using SPSS 19.0, and graphs were created using Origin 2022 software.

#### 2.4.1. Biological Accumulation Coefficient (BCF)


(1) $$\mathrm{BCF}=\frac{{\mathrm{HC}}_{\mathrm{corn}\;\mathrm{parts}}}{{\mathrm{HC}}_{\mathrm{soil}}}\;$$

Note: BCF means biological accumulation coefficient; HC means heavy metal content (mg/kg).

#### 2.4.2. Transfer Coefficient (TF)


(2) $${\mathrm{TF}}_{\mathrm{stem}\;\mathrm{and}\;\mathrm{leaf}}=\frac{{(\mathrm{Biomass}}_{\mathrm{Stem}}{\;\times \;\mathrm{HC}}_{\mathrm{stem}}{+\mathrm{Biomass}}_{\mathrm{leaf}}{\;\times \;\mathrm{HC}}_{\mathrm{leaf}})}{{(\mathrm{Biomass}}_{\mathrm{root}}{\;\times \;\mathrm{HC}}_{\mathrm{root}})}$$

(3) $${\mathrm{TF}}_{\mathrm{Grain}}=\frac{{(\mathrm{Biomass}}_{\mathrm{Grain}}{\;\times \;\mathrm{HC}}_{\mathrm{Grain}})}{{(\mathrm{Biomass}}_{\mathrm{Stem}}{\;\times \;\mathrm{HC}}_{\mathrm{stem}}{+\mathrm{Biomass}}_{\mathrm{leaf}}{\;\times \;\mathrm{HC}}_{\mathrm{leaf}})}\;$$

Note: TF means transfer coefficient.

#### 2.4.3. Heavy Metal Extraction Efficiency


(4) $${\mathrm{EF}}_{\mathrm{HM}}=\frac{\left(\sum {\;\mathrm{Biomass}}_{i}{\;\times \;\mathrm{HC}}_{i}\right)\;\times \;\mathrm{Planting}\;\mathrm{density}}{{(\mathrm{Bulk}\;\mathrm{density}\;\times \;\mathrm{Volume}\;\times \;\mathrm{HC}}_{\mathrm{soil}})}\;\times \;100\%$$

Note: EF_HM_ means heavy metal extraction efficiency; i means stem, leaf, root, and grain; planting density: 30 cm × 30 cm, 15 plants/m^2^; bulk density: 1.3 g/cm^3^; volume: 1 m^2^ × 0.2 m.

## 3. Results and Discussion

### 3.1. Distribution of Heavy Metal Content in Different Tissues of Corn

**Figure 2:**
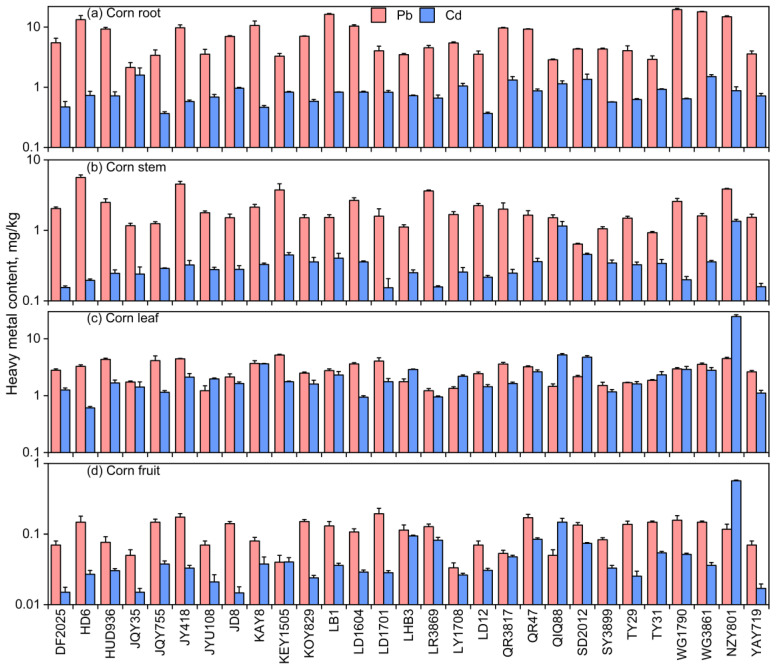
In (**a**) roots, (**b**) stems, (**c**) leaves, and (**d**) fruits of various corn varieties, the concentrations of Pb and Cd can be found.

Plants have the ability to take up heavy metals from the soil. Moreover, the distribution patterns of these heavy metals differ among various plant tissues. The physicochemical properties and heavy metal contents of the tested soil are shown in Table 1. As illustrated in Figure 2, the concentrations of Pb and Cd differ in various tissues of the same corn variety. A general pattern observed across different corn varieties is that the lowest concentrations of Pb (mean: 0.11 mg/kg) and Cd (mean: 0.06 mg/kg) are discovered in the corn fruits, while the most concentrated amount of Pb (7.44 mg/kg) is found in the roots and the highest concentration of Cd (2.83 mg/kg) is observed in the leaves. This suggests that heavy metals absorbed by plants tend to accumulate primarily in the roots and leaves, which is in line with the findings of Oh et al. [43], who reported that corn exhibits a strong accumulation capacity for Cu and Zn when used in the process of phytoremediating contaminated soils, with heavy metals predominantly distributed in the aerial parts of the plant, particularly the stems and leaves. The majority of plant species have a propensity to store metals within their roots. Conversely, merely a minor proportion of these metals is transported to the parts of the plant above the ground [44]. Furthermore, a previous study indicated that metals deposited from the atmosphere can accumulate in plants through foliar absorption, which may explain relatively high metal concentrations observed in the leaf tissues of corn [45,46].

For human health, heavy metal content within the consumable part of corn (the fruits) is of particular concern. Among the 29 corn varieties studied, the level of Pb found in the fruits of all varieties was below the maximum allowable limit set by China’s national standards regarding food contaminants in food safety, while Cd exceeded the limit in two varieties, QIQ88 and NZY801. The relatively low levels of heavy metals within the fruits suggest that corn can be applied to remediate soil contaminated by heavy metals through phytoremediation while still allowing for the safe harvest of crops for consumption. Xu et al. [47] also confirmed this conclusion, finding that when corn was grown in Cd-contaminated field soil, the levels of Cd within the tissues occurred in the sequence of sheath > root > lamina > stem > fruit, with Cd levels in the fruits being lower than the maximum allowable concentration level of Cd in cereals (0.1 mg/kg, dry weight) determined by the Ministry of Health of China (GB2762-2022) [48]. This may be due to the greater barriers to metal translocation from soil to fruits compared to leaves and stems [44]. In terms of heavy metal types, the uptake of Pb by the roots was substantially higher than that of Cd (*p* < 0.05), which may be associated with the bioavailability and specific characteristics of different metals, such as the tendency of Pb to be immobilized in the roots [49].

For the side-by-side comparisons among the 29 corn varieties we explored, the varieties with the peak concentrations of Pb and Cd within the root system were WG1790 and JQY35, respectively, and the varieties with the lowest levels were JQY35 and JQY755. In the stems, the varieties HD6 and SD2012, respectively, had the maximum and minimum concentrations of Pb, while NZY801 and LD1701 had the maximum and minimum Cd concentrations. For the leaves, KEY1505 and JYU108 exhibited the maximum and minimum Pb concentrations, and NZY801 and HD6 showed the maximum and minimum Cd concentrations. In the fruits, the varieties LD1701 and LY1708 had the maximum and minimum Pb concentrations, while NZY801 and JD8 had the maximum and minimum Cd concentrations. Notably, the roots of WG1790 and the leaves of NZY801 were the tissues with the highest Pb and Cd concentrations, respectively. After comparing the heavy metal contents across the different corn varieties, we observed significant differences in the uptake of Pb and Cd in the roots, stems, leaves, and fruits (Table S4). Moreover, specific varieties, such as WG1790 and NZY801, demonstrated a high-level capacity to enrich Pb and Cd in particular tissues (roots or leaves). These differences may be attributed to a variety of factors, including the genetic background of the varieties, their environmental adaptability, and the efficiency of the root system in absorbing heavy metals originating in the soil [50]. To further investigate the influence of corn varieties on heavy metal absorption and accumulation, we introduced the Enrichment Factor and Translocation Factor into the study. These parameters allow for a more holistic assessment of the differences in heavy metal uptake and translocation among the various corn varieties.

### 3.2. Enrichment and Transport Coefficients of Heavy Metals in Different Corn Varieties

The Bioconcentration Factor (BCF) and Translocation Factor (TF) serve as crucial indices for assessing a plant’s ability to take in and amass heavy metals or other pollutants from the environment. The BCF reflects the plant’s capacity to absorb a specific pollutant, while the TF reflect the plant’s ability to transport the absorbed pollutant from the roots up to the parts above the ground. A TF value greater than 1 typically suggests that the plant can effectively transport pollutants from the roots to the parts above the soil surface. The combined use of these two factors provides a more comprehensive assessment of a plant’s potential and effectiveness in remediating heavy-metal-contaminated soils [51,52,53,54]. Plants with both high BCF and TF values are particularly valuable for the removal of heavy metals from soil, as they can efficiently absorb and transport these pollutants.

**Figure 3:**
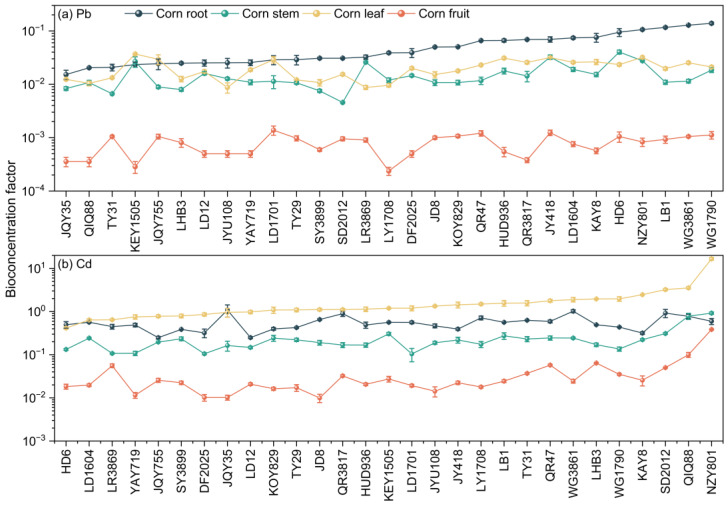
Bioconcentration factors of roots, stems, leaves, and fruits of different corn varieties for (**a**) Pb and (**b**) Cd.

The 29 corn varieties exhibit varying BCF and TF values for Pb and Cd. For BCF, the mean Pb and Cd values across the 29 varieties are 0.09 and 2.76, respectively, indicating that, overall, these varieties are more effective at absorbing Cd than Pb, which reflects the different bioconcentration capacities of different metals in plants. Among them, JQY35 and WG1790 represent the varieties with the lowest and highest BCF for Pb, respectively, with a difference of over fourfold. Cd, HD6, and NZY801 exhibit the lowest and highest BCF, respectively, with NZY801’s BCF being 17 times higher than that of HD6. The differences in bioconcentration factors across varieties are primarily related to their physiological structure, metal translocation mechanisms within the plant, and the root system’s ability to absorb metals [49,55]. These results indicate that WG1790 and NZY801 are the varieties with the strongest absorption capacities for Pb and Cd, respectively, and thus have a greater potential for remediating soils contaminated with Pb and Cd. As shown in Figure 3, among the different parts of the corn plant, the BCF for Pb is highest in the roots, while the BCF for Cd is highest in the leaves. This indicates that Pb tends to accumulate in the corn roots, whereas Cd tends to accumulate in the leaves. To further clarify the translocation of Pb and Cd within the corn plants, we also conducted a study on the TF.

For the overall TF of the corn plants, the mean TF values for Pb and Cd across the 29 varieties are 0.89 and 4.15, respectively. In the case of Pb, only 31.03% of the varieties have a TF greater than 1, indicating that most varieties are not effective at translocating Pb from the roots to the aboveground parts. This aligns with our conclusion in Section 3.1, where we found that Pb tends to concentrate in the roots for the tested corn varieties. This could be related to the gene expression in corn, where significant upregulation in the roots results in higher Pb bioconcentration [56]. KEY1505 and LB1 exhibit the highest and lowest TF values for Pb, respectively. For Cd, all varieties have a TF greater than 1, indicating that the 29 corn varieties can efficiently translocate Cd to the aboveground parts. NZY801 has the highest TF value for Cd, reaching 30.96, indicating that Cd is more easily translocated to the aerial parts than Pb, a conclusion also reached by Figlioli et al. [57]. The likely reason is that Pb has higher stability and is less easily translocated from the roots to the aerial parts, whereas Cd is more mobile and readily transported to the aboveground parts of the plant [50].

**Figure 4:**
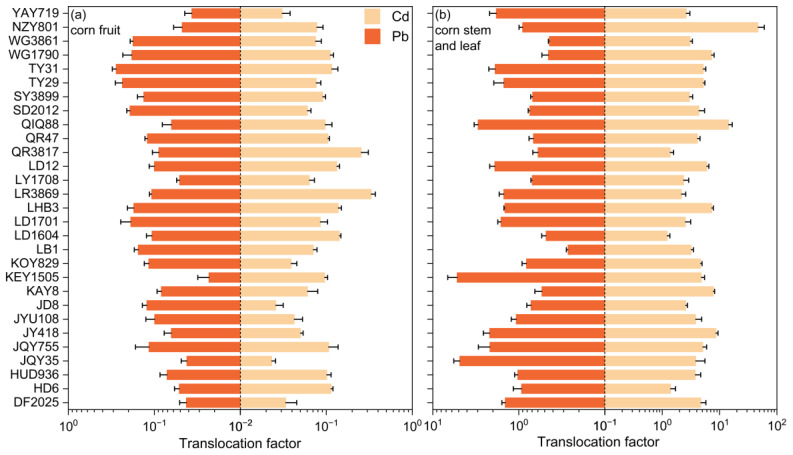
(**a**) Fruit translocation factors and (**b**) stem and leaf translocation factors of 29 corn varieties for Pb and Cd.

To further investigate which aboveground tissue exhibits a stronger capacity for heavy metal absorption, we also measured the TF for corn fruits and stems and leaves (as shown in Figure 4). The mean TF values for Pb and Cd in the fruits showed little difference (0.02 vs. 0.03), and the TF values for all varieties were less than 1, indicating that heavy metals are not easily translocated to the corn fruits. This further confirms that, for all the corn varieties studied, heavy metals are not readily transferred to the fruits, ensuring the safety of the edible parts of the corn plant and enabling the dual benefits of ecological remediation and economic development. The relatively high TF for Cd in the stems and leaves (mean: 4.08) suggests that Cd translocated to the aboveground parts primarily accumulates in the stems and leaves. Overall, when considering both BCF and TF, our study of the 29 corn varieties indicates that Pb is more likely to accumulate in the roots, Cd is more likely to accumulate in the leaves, and neither heavy metal is easily translocated to the corn fruits.

### 3.3. Effects of Amendment on Bioaccumulation Coefficient

In this study, we systematically evaluated the effects of different soil amendments (sepiolite, biochar, and the combination of sepiolite + biochar) on the bioaccumulation of lead (Pb) and cadmium (Cd) across various corn varieties. The results demonstrate significant variations in the bioconcentration factor (BCF) among corn varieties under different treatments.

**Figure 5:**
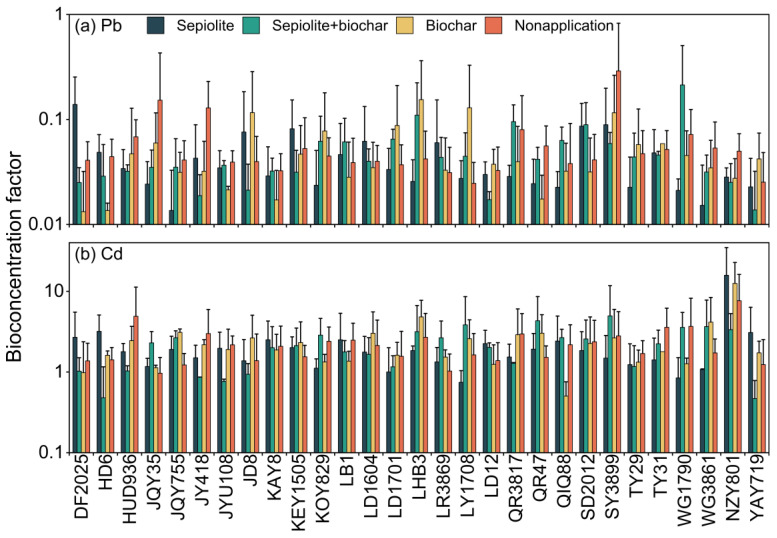
Effect of sepiolite, biochar, and combined amendments on the bioconcentration factor of (**a**) Pb and (**b**) Cd in different corn varieties.

As shown in Figure 5a, In the absence of any soil amendments (nonapplication), several corn varieties exhibited high Pb BCF values, with JQY35 and SY3899 displaying particularly elevated BCF values of 0.152 and 0.290, respectively. This indicates a strong inherent ability of these varieties to accumulate Pb, likely due to root structure characteristics and efficient ion exchange capacities, which facilitate Pb adsorption and retention within the plant tissues [58]. Under the combined treatment of sepiolite and biochar, the Pb BCF values were significantly reduced across most corn varieties. Notably, JY418, TY29, and DF2025 demonstrated BCF values as low as 0.018, 0.044, and 0.025, respectively. This substantial reduction in Pb uptake can be attributed to the synergistic effect of sepiolite and biochar, which enhances Pb immobilization in the soil. Biochar’s porous structure increases the soil’s cation exchange capacity, providing numerous sites for Pb ion adsorption, while sepiolite further enhances this process through its strong ion exchange and adsorption properties [59]. These findings suggest that, in Pb-contaminated soils, the use of high-Pb-accumulating corn varieties (e.g., JQY35 and SY3899) combined with sepiolite + biochar amendments could significantly reduce Pb mobility and accumulation within plant tissues, lowering the risk of Pb translocation in the food chain [60].

In Figure 5b, the BCF for cadmium (Cd) shows a different pattern. In terms of Cd accumulation, certain corn varieties, such as NZY801 and WG1790, demonstrated notably high Cd BCF values of 15.825 and 3.658 under nonapplication conditions, suggesting a strong potential for Cd accumulation. This elevated Cd BCF may be due to these varieties’ high affinity for Cd and efficient root uptake mechanisms [61]. However, when treated with sepiolite alone, certain varieties like QR47 and SY3899 exhibited a marked increase in Cd BCF values, reaching 4.325 and 4.963, respectively. This increase in Cd uptake is likely due to the ion exchange properties of sepiolite, which may enhance the bioavailability of Cd, making it more readily absorbed by the plant’s roots [62]. Sepiolite’s ion exchange can inadvertently increase Cd mobility, thereby enhancing Cd uptake in these varieties. For example, in DF2025 and TY31, the Cd BCF values decreased to 1.027 and 1.171, respectively, indicating a more effective reduction in Cd uptake compared to sepiolite or biochar alone. This could be due to biochar’s abundant adsorption sites, which decrease Cd mobility, and its alkaline nature, which raises soil pH and further limits Cd’s bioavailability to plants [63]. These results indicate that, in Cd-contaminated soils, combining high-Cd-accumulating corn varieties (such as NZY801 and WG1790) with sepiolite + biochar can effectively enhance Cd absorption and containment, thereby reducing Cd migration in the soil.

### 3.4. Effects of Amendment on Metal Translocation

**Figure 6:**
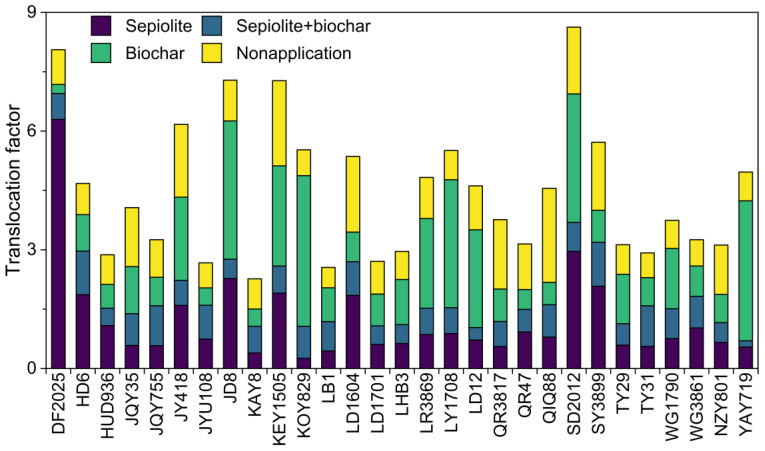
Effect of sepiolite, biochar, and combined treatments on the translocation factor of heavy metals in various corn varieties.

As illustrated in Figure 6, the Translocation Factor (TF), representing the ratio of heavy metal translocation from roots to aerial parts (e.g., shoots and leaves), varies across different treatments and corn varieties. Four treatments were applied: Sepiolite, Sepiolite + Biochar, Biochar, and Nonapplication (control). The overall trends indicate that the amendments, particularly biochar, play a significant role in reducing heavy metal translocation, as supported by several studies [64,65].

The results indicate that certain corn varieties (YAY719, LD12, JD8, and WG1790) consistently exhibited low TF values for Pb, particularly when treated with a combination of sepiolite and biochar. This treatment effectively reduced Pb mobility and kept it concentrated within the roots. For instance, YAY719 had a Pb TF value of 0.157 with the sepiolite + biochar combination, compared to 0.72 without treatment, showing an 80% reduction in Pb translocation. LD12 and JD8 also demonstrated significant reductions in Pb TF values with sepiolite + biochar, achieving values of 0.316 and 0.488, respectively, highlighting their effectiveness in Pb immobilization. The low TF values in these varieties under the sepiolite + biochar treatment suggest that this amendment creates an environment conducive to Pb retention in the root zone. Sepiolite contributes to Pb fixation through ion exchange, while biochar’s high cation exchange capacity and porous structure enhance metal adsorption, thereby reducing Pb bioavailability [66]. By keeping Pb confined to the roots, these varieties play a crucial role in stabilizing Pb in contaminated soils, limiting its upward movement and reducing the risk of exposure.

Similar trends were observed for Cd, with specific corn varieties showing minimal Cd translocation under the sepiolite + biochar treatment. Notable examples include the following: LD1701 with a Cd TF value of 0.77, down from 1.90 in the nonapplication group, indicating a 59% reduction. TY31 demonstrated a Cd TF value of 0.772 with sepiolite + biochar, compared to 4.10396 without amendments, reflecting an 81% reduction. These varieties effectively immobilize Cd within the root zone, which is critical for preventing the Cd contamination of edible plant parts. The combination of sepiolite and biochar likely contributes to this stabilization by modifying soil pH and providing abundant adsorption sites for Cd ions, thereby reducing their mobility [59]. By confining Cd within the roots, these varieties are well-suited for Cd stabilization, making them valuable for remediating Cd-polluted soils [67].

### 3.5. Selection of Optimal Materials and Corn Varieties

There are differences in the treatment of heavy metals Pb and Cd in the soil of corn varieties. The roots and leaves are the main accumulation sites of heavy metals, while the content of heavy metals in corn fruits is relatively low. Although the Pb content of most varieties reached the national standard, the Cd content of QIQ88 and NZY801 exceeded the standard. Specific varieties such as WG1790 and NZY801 showed strong heavy metal absorption capacity, which may be related to their genetic characteristics and root absorption efficiency. In terms of BCF and TF, there were significant differences among varieties, and the overall absorption capacity of Cd was stronger; while most varieties were not good at transporting Pb from the roots to aboveground parts, they could effectively transport Cd.

Regarding the remediation materials, the combination of sepiolite and biochar is the best soil amendment material because it shows significant effects in reducing the bioaccumulation of Pb and Cd in different corn varieties and limiting the migration of these heavy metals from the roots to the above-ground parts. This combination effectively reduces the bioavailability of Pb, enhances the absorption and containment of Cd, and changes soil pH while providing abundant adsorption sites for heavy metal ions, thereby reducing the accumulation of Pb and Cd in plant tissues and restricting their spread in the food chain, especially in corn varieties with high Pb and Cd accumulation. Therefore, it is considered the best choice among soil amendments.

When selecting phytoremediation varieties, it is necessary to comprehensively consider their absorption capacity, migration characteristics, and heavy metal content in fruits. The WG1790 variety is an ideal choice for the phytoremediation of Pb-contaminated soil due to its high Pb absorption capacity and low mobility. Although the NZY801 variety has a strong absorption capacity for Cd, the Cd content in its fruit exceeds the safety standard, so it is not suitable as an edible crop.

In summary, to achieve effective phytoremediation and ensure the safety of corn fruit, it is recommended to use a combination of sepiolite and biochar as a soil improvement material and select the WG1790 variety for planting. When selecting the optimal varieties and materials, multiple factors such as environmental adaptability, growth rate, yield, and resistance to other environmental pressures should also be considered [68]. In addition, it is recommended to conduct field experiments to verify the actual effects of these varieties and material combinations under different environmental conditions. These measures can maximize the efficiency of phytoremediation and ensure food safety. These results not only provide a scientific basis for soil pollution remediation, but also provide new strategies for sustainable agricultural development and environmental protection.

## 4. Conclusions

This study used biochar, sepiolite, and biochar–sepiolite composite amendments to evaluate the accumulation of Cd and Pb in soil with 29 corn varieties. The results show that the heavy metal content in corn fruits was of great concern, with Pb content not exceeding the standard and Cd exceeding the standard in two varieties. Certain varieties, such as WG1790 and NZY801, show strong heavy metal absorption capacity. There was significant variation (*p* < 0.05) in the accumulation of Pb and Cd in the roots, stems, leaves, and fruits of different corn varieties. The concentrations of Cd and Pb in corn fruits were the lowest (Pb: 0.11 mg/kg, Cd: 0.06 mg/kg). The ability of plants to absorb and accumulate heavy metals was evaluated using the bioaccumulation coefficient (BCF) and transfer coefficient (TF). Among the 29 corn varieties, the BCF and TF values for Pb and Cd were different, and the varietal differences were related to physiological structure, metal transport mechanisms, etc. The combination of biochar and sepiolite creates an environment conducive to the retention of heavy metals in the root zone, effectively reducing the risk of heavy metal contamination in the edible part. Considering the absorption capacity, migration characteristics, and heavy metal content in fruits of different crops, it is recommended to use sepiolite and biochar in combination as soil amendments and plant WG1790 varieties, while also considering environmental adaptability and other factors, as well as conducting field experiments to verify the effects. These results provide scientific evidence and new strategies for the selection of corn varieties and soil amendments.

## Figures and Tables

**Table 1:** Physicochemical properties and heavy metal content (dry weight) of tested soil.

Indicator	Sample Number	Average	Maximum	Minimum	Standard Deviation
pH	73	5.33	6.51	4.31	0.54
Organic Matter(%)	19	2.91	4.80	1.00	1.56
Total Nitrogen (as Nitrogen) (mg/kg)	19	919.99	1810.00	28.90	650.84
Total Phosphorus (as Phosphorus) (mg/kg)	19	1582.60	3730.00	659.00	830.02
Available Phosphorus (mg/kg)	19	20.65	37.80	6.20	9.14
Quick-acting Potassium (mg/kg)	19	126.83	240.00	50.60	53.95
Cation Exchange Capacity (cmol/kg)	19	11.39	27.50	3.60	6.56
Total Potassium (mg/kg)	19	16.00	27.20	6.90	5.70
Hg (mg/kg)	19	0.32	4.05	0.00	0.46
As (mg/kg)	19	20.39	303.00	0.34	31.28
Pb (mg/kg)	19	367.83	7761.00	2.26	650.11
Cu (mg/kg)	19	40.97	266.00	3.00	28.42
Ni (mg/kg)	19	49.70	672.00	3.00	52.73
Zn (mg/kg)	19	266.12	5490.00	18.00	481.49
Cd (mg/kg)	19	5.13	139.00	0.08	12.95
Cr (mg/kg)	19	116.18	498.00	1.00	75.56

## Data Availability

The original contributions presented in this study are included in the article/Supplementary Material. Further inquiries can be directed to the corresponding author.

## Supplementary Materials

The following supporting information can be downloaded at: https://www.mdpi.com/article/10.3390/toxics13020127/s1, Table S1: Corn variety screening results; Table S2: Fertilizer characteristics; Table S3: Measurement standard, standard solution, instrument used and detection limit; Table S4; Significant differences in Pb and Cd content in various parts of different corn varieties.

## References

[1] Komnitsas K., Modis K. (2006). Soil risk assessment of As and Zn contamination in a coal mining region using geostatisretics. Sci. Total Environ..

[2] Oladipo O.G., Olayinka A., Awotoye O.O.J.E., Biology E. (2014). Ecological impact of mining on soils of Southwestern Nigeria. Environ. Exp. Biol..

[3] Kaplan O., Ince M., Yaman M. (2011). Sequential extraction of cadmium in different soil phases and plant parts from a former industrialized area. Environ. Chem. Lett..

[4] Alam M., Snow E., Tanaka A. (2003). Arsenic and heavy metal contamination of vegetables grown in Samta village, Bangladesh. Sci. Total. Environ..

[5] Adriano D.C. (2001). Trace Elements in Terrestrial Environments: Biogeochemistry, Bioavailability, and Risks of Metals.

[6] Balkhair K.S., Ashraf M.A. (2016). Field accumulation risks of heavy metals in soil and vegetable crop irrigated with sewage water in western region of Saudi Arabia. Saudi J. Biol. Sci..

[7] Peana M., Pelucelli A., Medici S., Cappai R., Nurchi V.M., Zoroddu M.A. (2021). Metal Toxicity and Speciation: A Review. Curr. Med. Chem..

[8] Wang R.Y., Sang P.T., Guo Y.H., Jin P., Cheng Y.L., Yu H., Xie Y.F., Yao W.R., Qian H. (2023). Cadmium in food: Source, distribution and removal. Food Chem..

[9] Ahmad M., Soo Lee S., Yang J.E., Ro H.-M., Han Lee Y., Sik Ok Y. (2012). Effects of soil dilution and amendments (mussel shell, cow bone, and biochar) on Pb availability and phytotoxicity in military shooting range soil. Ecotoxicol. Environ. Saf..

[10] Park J.H., Lamb D., Paneerselvam P., Choppala G., Bolan N., Chung J.-W. (2011). Role of organic amendments on enhanced bioremediation of heavy metal(loid) contaminated soils. J. Hazard. Mater..

[11] Wang K.F., Peng N., Zhao P., Chen M.Q., Deng F.C., Yu X.L., Zhang D.Q., Chen J.F., Sun J.T. (2021). Effect of a low-cost and highly efficient passivator synthesized by alkali-fused fly ash and swine manure on the leachability of heavy metals in a multi-metal contaminated soil. Chemosphere.

[12] Zhao Q.-Y., Li X.-M., Yang Q., Chen C., Zhong Z.-Y., Zhong Y., Chen F., Chen X.-F., Wang X. (2018). Passivation of Simulated Pb-and Cd-Contaminated Soil by Applying Combined Treatment of Phosphate, Humic Acid, and Fly Ash. Huanjing Kexue.

[13] Gao L.-Y., Deng J.-H., Huang G.-F., Li K., Cai K.-Z., Liu Y., Huang F. (2019). Relative distribution of Cd2+ adsorption mechanisms on biochars derived from rice straw and sewage sludge. Bioresour. Technol..

[14] Huang F., Li K., Wu R.-R., Yan Y.-J., Xiao R.-B. (2020). Insight into the Cd2+ biosorption by viable Bacillus cereus RC-1 immobilized on different biochars: Roles of bacterial cell and biochar matrix. J. Clean. Prod..

[15] Liu Y., Tang J., Yuan J., Yao C., Hosoi K., Han Y., Yu S., Wei H., Chen G. (2020). Arsenite-induced downregulation of occludin in mouse lungs and BEAS-2B cells via the ROS/ERK/ELK1/MLCK and ROS/p38 MAPK signaling pathways. Toxicol. Lett..

[16] Zhou Z., Xu Z., Feng Q., Yao D., Yu J., Wang D., Lv S., Liu Y., Zhou N., Zhong M.-E. (2018). Effect of pyrolysis condition on the adsorption mechanism of lead, cadmium and copper on tobacco stem biochar. J. Clean. Prod..

[17] Xu C., Zhao J., Yang W., He L., Wei W., Tan X., Wang J., Lin A. (2020). Evaluation of biochar pyrolyzed from kitchen waste, corn straw, and peanut hulls on immobilization of Pb and Cd in contaminated soil. Environ. Pollut..

[18] Chamon A., Gerzabek M., Mondol M., Ullah S., Rahman M., Blum W.E.H. (2005). Influence of soil amendments on heavy metal accumulation in crops on polluted soils of Bangladesh. Commun. Soil Sci. Plant Anal..

[19] Angelova V., Akova V., Artinova N., Ivanov K.I. (2013). The effect of organic amendments on soil chemical characteristics. Bulg. J. Agric. Sci..

[20] Xu C., Yang W., Zhu L., Juhasz A.L., Ma L.Q., Wang J., Lin A. (2017). Remediation of polluted soil in China: Policy and technology bottlenecks. Environ. Sci. Technol..

[21] Wang Y.Y., Liu Y.D., Zhan W.H., Zheng K.X., Wang J.N., Zhang C.S., Chen R.H. (2020). Stabilization of heavy metal-contaminated soils by biochar: Challenges and recommendations. Sci. Total Environ..

[22] Van Poucke R., Ainsworth J., Maeseele M., Ok Y.S., Meers E., Tack F. (2018). Chemical stabilization of Cd-contaminated soil using biochar. Appl. Geochem..

[23] Puga A.P., Melo L.C.A., de Abreu C.A., Coscione A.R., Paz-Ferreiro J.J.S., Research T. (2016). Leaching and fractionation of heavy metals in mining soils amended with biochar. Soil Tillage Res..

[24] Puga A., Abreu C., Melo L., Beesley L. (2015). Biochar application to a contaminated soil reduces the availability and plant uptake of zinc, lead and cadmium. J. Environ. Manag..

[25] Liu S., Lu Y., Yang C., Liu C., Ma L., Dang Z. (2017). Effects of modified biochar on rhizosphere microecology of rice (*Oryza sativa* L.) grown in As-contaminated soil. Environ. Sci. Pollut. Res..

[26] Lan J.R., Zhang S.S., Dong Y.Q., Li J.H., Li S.Y., Feng L., Hou H.B. (2021). Stabilization and passivation of multiple heavy metals in soil facilitating by pinecone-based biochar: Mechanisms and microbial community evolution. J. Hazard. Mater..

[27] Li Y., Huang Y., Wei L., Huang L., Huang Q., Xu G., Liu Z. (2017). Impacts of biochar application on amelioration of heavy metal-polluted soil and maize growth. J. Agro-Environ. Sci..

[28] Zhang T., He Y. (2016). Environmental adsorbing material prepared by sepiolite. Non-Met. Mines.

[29] Lui R., Ji Z., Tan J., Wang J., Zhang J., Liao X. (2017). Advances in preparation and photocatalytic properties of sepiolite-based metal oxide compounds. Mater. Rep..

[30] Qin X., Liu Y., Huang Q., Liu Y., Zhao L., Xu Y. (2019). In-Situ Remediation of Cadmium and Atrazine Contaminated Acid Red Soil of South China Using Sepiolite and Biochar. Bull. Environ. Contam. Toxicol..

[31] Hou R.J., Zhu B.Y., Wang L.W., Gao S.J., Wang R., Hou D.Y. (2024). Mechanism of clay mineral modified biochar simultaneously immobilizes heavy metals and reduces soil carbon emissions. J. Environ. Manag..

[32] Deng J., Xu Z., Dai Y., Zhong J., Shi F., Wang J., Li W., Li Y., Huang Y., Zhang Y. (2025). Screening of practical low-accumulating crops in cadmium-polluted farmland: A field survey and field trail in Guangdong Province, China. J. Clean. Prod..

[33] Lin K., Zeng M., Williams D.V., Hu W., Shabala S., Zhou M., Cao F. (2022). Integration of Transcriptome and Metabolome Analyses Reveals the Mechanistic Basis for Cadmium Accumulation in Maize. iScience.

[34] China, Ministry of Agriculture of the People’s Republic of China, Soil Testing—Part 6: Determination of Soil Organic Matter China. https://www.cnemc.cn/jcgf/trhj/201711/t20171107_647331.shtml.

[35] China, Ministry of Agriculture of the People’s Republic of China, Determination of Copper, Zinc, Lead, Nickel, Chromium in Soil and Sediments by Flame Atomic Absorption Spectrophotometry. China. https://www.mee.gov.cn/ywgz/fgbz/bz/bzwb/jcffbz/201905/t20190513_702667.shtml.

[36] General Administration of Quality Supervision, General Administration of Quality Supervision, Inspection and Quarantine of the People’s Republic of China, Soil Quality—Determination of Lead and Cadmium by Graphite Furnace Atomic Absorption Spectrophotometry. China. https://www.mee.gov.cn/ywgz/fgbz/bz/bzwb/jcffbz/199805/t19980501_82029.shtml.

[37] General Administration of Quality Supervision, General Administration of Quality Supervision, Inspection and Quarantine of the People’s Republic of China, Soil Quality—Determination of Total Mercury, Total Arsenic, Total Lead by Atomic Fluorescence Spectrometry. China. https://www.mee.gov.cn/ywgz/fgbz/bz/bzwb/jcffbz/201312/t20131203_264304.htm.

[38] China, Ministry of Agriculture of the People’s Republic of China, Determination of Total Mercury in Soil and Sediments by Catalytic Thermal Decomposition-Cold Atomic Absorption Spectrophotometry. China. https://www.mee.gov.cn/ywgz/fgbz/bz/bzwb/jcffbz/201801/t20180108_429304.shtml.

[39] China, Ministry of Agriculture of the People’s Republic of China, Determination of Soil Cation Exchange Capacity by Cobalt Hexamine Chloride Extraction-Photometric Method. China. https://www.mee.gov.cn/ywgz/fgbz/bz/bzwb/jcffbz/201712/t20171220_428288.shtml.

[40] China, State Forestry Administration of the People’s Republic of China, Determination of Total Potassium in Forest Soils. China. https://rif.caf.ac.cn/info/1106/3673.htm.

[41] China, Ministry of Agriculture of the People’s Republic of China, Soil Testing—Part 24: Determination of Total Nitrogen in Soil by Automatic Kjeldahl Method. China. https://nynct.guizhou.gov.cn/ztzl/ncpzlaq/201705/t20170519_24406260.html.

[42] China, Ministry of Agriculture of the People’s Republic of China, Soil—Determination of Total Phosphorus by Alkaline Fusion-Molybdenum Antimony Photometric Method. China. https://www.mee.gov.cn/ywgz/fgbz/bz/bzwb/jcffbz/201112/t20111213_221313.shtml.

[43] Deng C.X., Wen J.J., Li Z.W., Luo N.L., Huang M., Yang R. (2018). Passivating effect of dehydrated sludge and sepiolite on arsenic contaminated soil. Ecotoxicol. Environ. Saf..

[44] Oh K., Li T., Cheng H., Hu X., Lin Q., Xie Y. A Primary Study on Assessment of Phytoremediation Potential of Biofuel Crops in Heavy Metal Contaminated soil. Proceedings of the International Conference on Sustainable Energy and Environmental Engineering (ICSEEE 2012).

[45] Sun F.F., Wang F.H., Wang X., He W., Wen D., Wang Q.F., Liu X.X. (2013). Soil threshold values of total and available cadmium for vegetable growing based on field data in Guangdong province, South China. J. Sci. Food Agric..

[46] Gan Y., Wang L., Yang G., Dai J., Wang R., Wang W. (2017). Multiple factors impact the contents of heavy metals in vegetables in high natural background area of China. Chemosphere.

[47] Chen Z.-L., Huang L., Zhou C.-Y., Zhong S.-X., Wang X., Dai Y., Jiang X.-L. (2017). Characteristics and Evaluation of Heavy Metal Pollution in Vegetables in Guangzhou. Huanjing Kexue.

[48] Xu W., Lu G., Dang Z., Liao C., Chen Q., Yi X. (2013). Uptake and Distribution of Cd in Sweet Maize Grown on Contaminated Soils: A Field-Scale Study. Bioinorg. Chem. Appl..

[49] China, National Health Commission of the People’s Republic of China. National Standards for Food Safety Limits of contaminants in food. 2022. https://www.samr.gov.cn/.

[50] Atta M.I., Zehra S.S., Ali H., Ali B., Abbas S.N., Aimen S., Sarwar S., Ahmad I., Hussain M., Al-Ashkar I. (2023). Assessing the effect of heavy metals on maize (*Zea mays* L.) growth and soil characteristics: Plants-implications for phytoremediation. Peerj.

[51] Ren C., Xiao J.-H., Li J.-T., Du Q.-Q., Zhu L.-W., Wang H., Zhu R.-Z., Zhao H.-Y. (2022). Accumulation and Transport Characteristics of Cd, Pb, Zn, and As in Different Maize Varieties. Huanjing Kexue.

[52] Sanjose I., Navarro-Roldan F., Montero Y., Ramirez-Acosta S., Javier Jimenez-Nieva F., Infante-Izquierdo M.D., Polo-Avila A., Francisco Munoz-Rodriguez A. (2022). The Bioconcentration and the Translocation of Heavy Metals in Recently Consumed Salicornia ramosissima J. Woods in Highly Contaminated Estuary Marshes and Its Food Risk. Diversity.

[53] Usman K., Al-Ghouti M.A., Abu-Dieyeh M.H. (2019). The assessment of cadmium, chromium, copper, and nickel tolerance and bioaccumulation by shrub plant *Tetraena qataranse*. Sci. Rep..

[54] Islam M.D., Hasan M.M., Rahaman A., Haque P., Islam M.S., Rahman M.M. (2020). Translocation and bioaccumulation of trace metals from industrial effluent to locally grown vegetables and assessment of human health risk in Bangladesh. SN Appl. Sci..

[55] Chen Q., Shen Y., Fang Y., Yan J., Li P., Zhang K. (2014). Heavy metals pollution risk and characteristics of plant accumulation along Zihu River. Trans. Chin. Soc. Agric. Eng..

[56] Li J.-K., Zhang D., Zhou P., Liu Q.-L. (2018). Assessment of Heavy Metal Pollution in Soil and Its Bioaccumulation by Dominant Plants in a Lead-Zinc Mining Area, Nanjing. Huan Jing Ke Xue Huanjing Kexue.

[57] Gao J., Zhang Y., Lu C., Peng H., Luo M., Li G., Shen Y., Ding H., Zhang Z., Pan G. (2015). The development dynamics of the maize root transcriptome responsive to heavy metal Pb pollution. Biochem. Biophys. Res. Commun..

[58] Figlioli F., Sorrentino M.C., Memoli V., Arena C., Maisto G., Giordano S., Capozzi F., Spagnuolo V. (2019). Overall plant responses to Cd and Pb metal stress in maize: Growth pattern, ultrastructure, and photosynthetic activity. Environ. Sci. Pollut. Res..

[59] Liu L., Li J., Yue F., Yan X., Wang F., Bloszies S., Wang Y. (2018). Effects of arbuscular mycorrhizal inoculation and biochar amendment on maize growth, cadmium uptake and soil cadmium speciation in Cd-contaminated soil. Chemosphere.

[60] Hamid Y., Tang L., Hussain B., Usman M., Gurajala H.K., Rashid M.S., He Z., Yang X. (2020). Efficiency of lime, biochar, Fe containing biochar and composite amendments for Cd and Pb immobilization in a co-contaminated alluvial soil. Environ. Pollut..

[61] Haider F.U., Virk A.L., Rehmani M.I.A., Skalicky M., Ata-ul-Karim S.T., Ahmad N., Soufan W., Brestic M., Sabagh A.E.L., Cai L. (2022). Integrated Application of Thiourea and Biochar Improves Maize Growth, Antioxidant Activity and Reduces Cadmium Bioavailability in Cadmium-Contaminated Soil. Front. Plant Sci..

[62] Sun Y., Xu Y., Xu Y., Wang L., Liang X., Li Y. (2016). Reliability and stability of immobilization remediation of Cd polluted soils using sepiolite under pot and field trials. Environ. Pollut..

[63] Zhou C., Song X., Wang Y., Wang H., Ge S. (2022). The sorption and short-term immobilization of lead and cadmium by nano-hydroxyapatite/biochar in aqueous solution and soil. Chemosphere.

[64] Alaboudi K.A., Ahmed B., Brodie G. (2019). Effect of biochar on Pb, Cd and Cr availability and maize growth in artificial contaminated soil. Ann. Agric. Sci..

[65] Rafique M., Ortas I., Rizwan M., Sultan T., Chaudhary H.J., Isik M., Aydin O. (2019). Effects of Rhizophagus clarus and biochar on growth, photosynthesis, nutrients, and cadmium (Cd) concentration of maize (Zea mays) grown in Cd-spiked soil. Environ. Sci. Pollut. Res..

[66] Maharlouei Z.D., Fekri M., Saljooqi A., Mahmoodabadi M., Hejazi M. (2021). Effect of modified biochar on the availability of some heavy metals speciation and investigation of contaminated calcareous soil. Environ. Earth Sci..

[67] Haider F.U., Farooq M., Naveed M., Cheema S.A., ul Ain N., Salim M.A., Cai L., Mustafa A. (2022). Influence of biochar and microorganism co-application on stabilization of cadmium (Cd) and improved maize growth in Cd-contaminated soil. Front. Plant Sci..

[68] Kumar Yadav K., Gupta N., Kumar A., Reece L.M., Singh N., Rezania S., Ahmad Khan S. (2018). Mechanistic understanding and holistic approach of phytoremediation: A review on application and future prospects. Ecol. Eng..

