# Supplementary material for: Effects of Biochar and Sepiolite on Pb and Cd Dynamics in Contaminated Soil with Different Corn Varieties
Source: Toxics. 2025 Feb 9;13(2):127. doi: 10.3390/toxics13020127 (PMC11860827; doi:10.3390/toxics13020127)
Supplement: Supplementary file 1 [file toxics-13-00127-s001.zip › toxics-3442408-supplementary.pdf]

Supplementary information for

Effects of Biochar and Sepiolite on Pb and Cd Dynamics in Contaminated Soil with Different Corn Varieties

Table S1 Corn variety screening results

No.	Source of Variety	Variety	Quantity (pieces)	Environmental Characteristics	Pollution Characteristics
1	Main varieties promoted by the Agriculture Technology Promotion Center of Lancang County in the past 3 years	Hongdan No. 6, Ludan No. 12, Jinyin 418, Xikang 18, Kangnong No. 2, Dika 007, Shangdan 2012, Wugu 1790, Zhengda 615, Wugu 3861, Longbai No. 1, Xianda 901, Longdan 1604, Longyu 1708, Yaoyu 4126, Ruidan 26, Dingdan 6789, Zhengda 719	18	Elevation of 1600-2090 m in Menglang Town, Lancang County; subtropical mountain monsoon climate; soil mainly consists of red clay and rocky types; pH value ranges from 3.92 to 8.09, overall acidic	Soil in the agricultural area exceeds limits for 8 heavy metals: mercury, arsenic, lead, copper, nickel, zinc, chromium, and cadmium; primarily contaminated by cadmium and lead
2	Main varieties sold in local stores based on field survey	Qingsui 47, Qingsui 119, Qingsui 3817, Jixiangyu 2199, Jingdian 8, Qingqing 009, Qingqing 515, Tianyan 8, Tianyan 29, Tianyan 31, Kebe 1409, Qiuhong 1, Qiuhong 88, Funongyu 1, Longrui 3869, Ziyu 88, Kangyu 008, Yiyu	60	Elevation of 1600-2090 m in Menglang Town, Lancang County; subtropical mountain monsoon climate; soil mainly consists of red clay and rocky types; pH	Soil in the agricultural area exceeds limits for 8 heavy metals: mercury, arsenic, lead, copper, nickel, zinc,

		8, Yayu 719, Yayu 1281, Jindan 208, Kenyu 1505, Jinyu 98, Jinyu 108, Shangyu 3899, Huidan 936, Xinzhuangyu 801, Kongyu 829, Dandan 908, Kebe 1409, Ludan No. 12, Jinyin 418, Fengdeng 2025, Hongdan No. 6, Xikang 18, Kangnong No. 2, Dika 007, Shangdan 2012, Shangdan 3721, Shangdan 365, Wugu 1790, Zhengda 615, Wugu 3861, Longbai No. 1, Xianda 901, Longdan 1604, Longdan 1609, Longdan 1701, Longyu 1708, Yaoyu 4123, Yaoyu 4126, Ruidan 26, Dingdan 6789, Zhengda 719, Longhuangbai No. 3, Jingdian 8, Yuanyu 093, Yunrui 119, Yunrui 668, Yudan 8		value ranges from 3.92 to 8.09, overall acidic	chromium, and cadmium; primarily contaminated by cadmium and lead
3	Low accumulation varieties selected from “the Soil Pollution Remediation Project in Zhehai Town, Huize County”	Luodan 566, Xuanhui No. 7, Diwo No. 2, Xianyu 696, Xuanhuangdan No. 5, Huaxing No. 7	6	Elevation of approximately 2050 m in the project area of Zhehai Town, Huize County; southern temperate monsoon climate; soil mainly consists of red soil and red clay; pH value ranges from 4.17 to 7.77, overall acidic to slightly acidic	Agricultural soil in the project area is simultaneously contaminated with Cd, Pb, Zn, Hg, As, Cu, and Ni. Cd is heavily polluted, Pb and Zn are primarily heavily polluted,

					while others are moderately to lightly polluted
4	Low accumulation varieties selected from “the Soil Pollution Control and Remediation Demonstration Project in Lanping County”	Xuanhuangdan No. 5, Quchan 11, Jingdian 4, Ludan 16, Chengxin 1, Chengxin 5, Longsheng 16, Qiangsheng 103, Yunrui 8, Huidan 4, Ludan 7, Xidan 8, Ludan 2, Ludan 6, Qiushuo 6	15	Elevation above 2240 m in Jinding Town, Lanping County; subtropical mountain monsoon climate; soil mainly consists of purple soil; pH value ranges from 4.42 to 8.79, overall slightly acidic	Soil in the project area is severely contaminated with Pb, Cd, and Zn, mainly exhibiting composite pollution from Cd and Pb
5	Low accumulation varieties selected from “the Soil Pollution Control and Remediation Technology Application Pilot Project in the Haojiahe River Basin, Mouding County”	Luodan 299, Datian 006, Enyu No. 8, Zhuoyu 299, Xianyu 696, Jinnong 109, Shengyu 6, Luodan 297, Jinqiuyuyu 35, Wugu 3861, Shengyu 8, Luodan 299, Kenyu 1505	13	Elevation of 1678-1880 m in Xinqiao Town, Mouding County; subtropical monsoon climate; soil mainly consists of purplish-red sandy mudstone; pH value ranges from 6.56 to 8.50, overall alkaline	Main contaminants in agricultural soil along the Haojiahe River are Cd and Cu

Note： Fengdeng 2025 (DF2025) , Hongdan 6 (HD6), Huidan 936 (HUD936), Jinqiu Yu 35 (JQY35), Jinqiu Yu 755 (JQY755), Jinyi 418 (JY418), Jinyu 108 (JYU108), Jingdian 8 (JD8), Kangyu 8 (KAY8), Kenyu 1505 (KEY1505), Kongyu 829 (KOY829), Longbai 1 (LB1), Longdan 1604 (LD1604), Longdan 1701 (LD1701), Longhuangbai

3 (LHB3), Longrui 3869 (LR3869), Longyu 1708 (LY1708), Ludan 12 (LD12), Qunrui 3817 (QR3817), Qunrui 47 (QR47), Qiuxing 88 (QIQ88), Shangdan 2012 (SD2012), Shangyu 3899 (SY3899), Tianyan 29 (TY29), Tianyan 31 (TY31), Wugu 1790 (WG1709), Wugu 3861 (WG3861), Xinzhongyu 801 (NZY801), and Yayu 719 (YAY719).

Table S2: Fertilizer characteristics

	N[a]	K[a]	P[a]	Cd (mg/kg)	Pb (mg/kg)	Fertilizer application (kg)[b]
Compound fertilizer	1.00	1.30	0.40	ND	ND	100
Organic fertilizer	1.00	0.50	0.50	1.80	ND	500

a: Fertilizer allocation ratio

b: The application amount of fertilizer was obtained from interviews with local villagers in the experiment

ND means below the limit of detection.

Table S3: Measurement standard, standard solution, instrument used and detection limit

	Equipment used	Standard solution	Detection limit	Reference
Organic Matter	Oil bath(DU-30); Burette	Potassium dichromate, sulfuric acid, ammonium ferrous sulfate or ferrous sulfate, o-phenanthroline	1.0 g/kg	[1]
Cu	Temperature-controlled heating plate (SD46-1); Flame atomic absorption spectrometer (280FS)	Hydrochloric acid, nitric acid, perchloric acid, hydrofluoric acid, Cu standard solution	1.0 mg/kg	[2]
Cr	Temperature-controlled heating plate (SD46-1); Flame atomic absorption spectrometer (280FS)	Hydrochloric acid, nitric acid, perchloric acid, hydrofluoric acid, Cr standard solution	4.0 mg/kg	[2]
Ni	Temperature-controlled heating plate (SD46-1); Flame atomic absorption spectrometer (280FS)	Hydrochloric acid, nitric acid, perchloric acid, hydrofluoric acid, Ni standard solution	3.0 mg/kg	[2]
Zn	Temperature-controlled heating plate (SD46-1); Flame atomic absorption spectrometer (280FS)	Hydrochloric acid, nitric acid, perchloric acid, hydrofluoric acid, Zn standard solution	1.0 mg/kg	[2]
Pb	Temperature-controlled heating plate (SD46-1); Flame atomic absorption spectrometer (280FS)	Hydrochloric acid, nitric acid, perchloric acid, hydrofluoric acid, Pb standard solution	0.1 mg/kg	[3]
Cd	Temperature-controlled heating plate (SD46-1); Flame atomic absorption spectrometer (280FS)	Hydrochloric acid, nitric acid, perchloric acid, hydrofluoric acid, Cd standard solution	0.01 mg/kg	[3]
As	Atomic fluorescence spectrometer	Hydrochloric acid, nitric acid, potassium	0.01 mg/kg	[4]

	(AFS-8220); Water bath (HWS-28)	hydroxide, potassium borohydride, thiourea, ascorbic acid, As standard solution		
Hg	Mercury analyzer (DMA-80)	Potassium dichromate, nitric acid, Hg standard solution	0.2 μg/kg	[5]
Cation Exchange Capacity	Constant temperature shaker (GWQ-12B); Centrifuge (TD5M); Spectrophotometer (TU-1810)	Cobalt hexamine chloride	0.8 cmol/kg	[6]
Total Potassium	Temperature-controlled heating plate (SD46-1); Flame atomic absorption spectrometer (280FS)	Hydrochloric acid, nitric acid, perchloric acid, hydrofluoric acid, potassium standard solution	-	[7]
Total Nitrogen	Automatic nitrogen analyzer (KDN); Digestion instrument	Sulfuric acid, sodium hydroxide, boric acid, octanol, sulfuric acid or hydrochloric acid standard titration solution, methyl red, bromocresol green, potassium permanganate, reducing iron powder, potassium sulfate, copper sulfate pentahydrate, selenium powder	-	[8]
Total Phosphorus	Spectrophotometer (TU-1810); Muffle furnace (SX2-12-10N); Centrifuge (TD5M)	Sulfuric acid, sodium hydroxide, anhydrous ethanol, nitric acid, ascorbic acid, ammonium molybdate, potassium antimony tartrate, phosphorus standard solution, 2,4-dinitrophenol	10.0 mg/kg	[9]

Table S4: Significant differences in Pb and Cd content in various parts of different corn varieties.

Corn varieties	Roots		Stem		Leaf		Fruit	
	Pb	Cd	Pb	Cd	Pb	Cd	Pb	Cd
DF2025	ef[a]	kl	efghi	i	fg	hij	jkl	mn
HD6	c	ghijk	a	hi	ef	j	bcde	ijklm
HUD936	d	ghijk	def	ghi	b	fghi	ijk	hijk
JQY35	j	a	klmn	ghi	ijk	ghij	klm	mn
JQY755	ghi	l	jklm	fgh	bc	hij	bcde	ghi
JY418	d	jkl	b	efg	b	defg	ab	hijk
JYU108	ghi	ghijk	ghij	fgh	k	efgh	jkl	klmn
JD8	e	efg	ijkl	fgh	hi	fghi	cdef	n
KAY8	d	kl	defgh	efg	cde	c	ijk	ghi
KEY1505	ghi	fghij	c	cd	a	fghi	lm	fgh
KOY829	e	jkl	ijkl	cdef	gh	fghi	bcd	jklmn
LB1	b	fghij	ijkl	cde	fg	def	defg	ghij
LD1604	d	fghij	d	cdef	cde	ij	ghi	hijkl
LD1701	fgh	fghij	hijkl	i	bcd	fghi	a	hijkl
LHB3	ghi	ghijk	klmn	ghi	ijk	cd	fgh	c
LR3869	fg	hijk	c	i	k	ij	defg	d
LY1708	ef	def	hijk	fghi	jk	defg	m	ijklmn
LD12	ghi	l	defg	hi	gh	ghij	jkl	hijk
QR3817	d	bcd	fghi	ghi	cde	fghi	jklm	efg
QR47	d	efghi	hijk	cdef	ef	de	abc	cd
QIQ88	hi	cde	ijkl	b	jk	b	klm	b
SD2012	fgh	abc	n	c	hi	b	defg	d
SY3899	fgh	jkl	lmn	defg	jk	hij	hij	hijk
TY29	fgh	ijkl	ijklm	efg	ijk	fghi	defg	jklmn
TY31	ghi	efgh	mn	efg	ij	def	bcde	e

WG1709	a	ijk	de	hi	fg	cd	bcd	ef
WG3861	a	ab	hijkl	cdef	de	cde	bcde	ghij
NZY801	bc	efghi	c	a	b	a	efg	a
YAY719	ghi	ghijk	ijkl	i	gh	ij	jkl	lmn

a: Differences without the same letter are considered significant

Reference:

1. China, Ministry of Agriculture of the People's Republic of China, Soil Testing - Part 6: Determination of Soil Organic Matter China.
2. China, Ministry of Agriculture of the People's Republic of China, Determination of Copper, Zinc, Lead, Nickel, Chromium in Soil and Sediments by Flame Atomic Absorption Spectrophotometry. China.
3. General Administration of Quality Supervision, General Administration of Quality Supervision, Inspection and Quarantine of the People's Republic of China, Soil Quality - Determination of Lead and Cadmium by Graphite Furnace Atomic Absorption Spectrophotometry. China.
4. General Administration of Quality Supervision, General Administration of Quality Supervision, Inspection and Quarantine of the People's Republic of China, Soil Quality - Determination of Total Mercury, Total Arsenic, Total Lead by Atomic Fluorescence Spectrometry. China.
5. China, Ministry of Agriculture of the People's Republic of China, Determination of Total Mercury in Soil and Sediments by Catalytic Thermal Decomposition-Cold Atomic Absorption Spectrophotometry. China.
6. China, Ministry of Agriculture of the People's Republic of China, Determination of Soil Cation Exchange Capacity by Cobalt Hexamine Chloride Extraction-Photometric Method. China.
7. China, State Forestry Administration of the People's Republic of China, Determination of Total Potassium in Forest Soils. China.
8. China, Ministry of Agriculture of the People's Republic of China, Soil Testing - Part 24: Determination of Total Nitrogen in Soil by Automatic Kjeldahl Method. China.
9. China, Ministry of Agriculture of the People's Republic of China, Soil - Determination of Total Phosphorus by Alkaline Fusion-Molybdenum Antimony Photometric Method. China.
